# A neurobehavioral therapy approach to the rehabilitation and support of persons with brain injury: Practice-based evidence from a UK charitable rehabilitation provider

**DOI:** 10.3389/fresc.2022.902702

**Published:** 2022-07-25

**Authors:** Rudi Coetzer, Sara da Silva Ramos

**Affiliations:** ^1^Clinical Department, The Disabilities Trust, Wakefield, United Kingdom; ^2^School of Human and Behavioural Sciences, Bangor University, Bangor, United Kingdom; ^3^Faculty of Medicine, Health and Life Sciences, Swansea University, Swansea, United Kingdom; ^4^Faculty of Health and Medical Science, University of Surrey, Guildford, United Kingdom

**Keywords:** holistic neurorehabilitation, psychological therapies, service development, interventions, neurobehavioral, outcomes

## Abstract

**Background:**

The treatment and rehabilitation for people with acquired brain injury is continually evolving, with increasing recognition of the importance of approaches that adopt a multi-disciplinary biopsychosocial perspective focused on improving adjustment, social participation, and wellbeing. However, there is significant variability as to how such approaches are delivered, across the various stages of recovery, rehabilitation settings, and within different healthcare systems.

**Objective:**

This paper had three aims. The first was to describe the neurobehavioral therapy (NBT) approach to brain injury rehabilitation adopted in our charitable organization. The second aim was to report how the NBT approach evolved in response to changes in referral patterns, and patient needs within a broader, longer-term clinical pathway. The third aim was to assess the effectiveness of the NBT approach by analyzing outcome data.

**Methods:**

Retrospective analyses of standardized outcome data were completed to investigate the effectiveness of our approach. Case vignettes are provided to illustrate the key components of the approach.

**Results:**

Outcome data suggested that the approach is effective in delivering positive outcomes for patients. Furthermore, the data show differences in presentation between three clinical streams (restoration, compensation, and scaffolding) within the NBT approach.

**Conclusions:**

This paper describes the adaption of the ‘traditional' neurobehavioral approach to brain injury rehabilitation into a model of delivery that can benefit a more diverse range of people living with the heterogenous and long-term consequences of brain injury.

## Introduction

As opposed to the *treatment* of brain injuries, which potentially goes back almost 3,000 years when considering the Edwin Smith papyrus (1), the *rehabilitation* of neurological patients is a much younger discipline (2). Furthermore, in this historical context, the treatment of a person with a brain injury was perhaps best conceptualized as early forms of “neurosurgical” intervention to manage the physical integrity of acute patients. In contrast, rehabilitation tends to focus more on post-acute and long-term multi-disciplinary interventions to improve outcomes for patients, and often contains a substantial psychological component.

The first attempts to rehabilitate (mostly post-acute) patients during the mid-nineteenth century, relied more on a Hebbian (3) approach of repetition and re-learning of lost skills after brain injury to re-establish damaged neural networks. Today, this would be described as a restorative approach to rehabilitation (4). Since that time, the wider field of neurorehabilitation has grown, and several new approaches to rehabilitation have been developed. Wilson (5) provided one of the first overviews of the main theoretical underpinnings of modern neuropsychological rehabilitation: the Cognitive Neuropsychology model; the Cognitive Rehabilitation model, the Combined model and the Holistic model.

More recently, Wilson et al. (2) described much more comprehensively the historical development and main epochs of neuropsychological rehabilitation, including for example the seminal work of Donald Hebb, A. R. Luria and Oliver Zangwill, among others. Wilson concludes the overview by pointing out that in clinical practice several theories and models are needed to best serve the complex combinations of cognitive, affective, and behavioral impairments and associated disability clients present with.

The Brain Injury Rehabilitation Trust (The Disabilities Trust, UK) is the number one brain injury rehabilitation charity in the United Kingdom. The Trust's first rehabilitation center, Thomas Edward Mitton House, established in 1991, had a strong behavioral ethos, which evolved to incorporate aspects of cognitive theory fundamental to human learning, informing all rehabilitation activities (6).

At present the Trust has two hospitals, seven assessment and rehabilitation centers and eight continuing brain injury rehabilitation units. Since offering specialist brain injury rehabilitation in 1991, there has been an ongoing emphasis on shaping the rehabilitation provided to patients around a neurobehavioral approach. Originally, the approach was based on a “purer” behavioral model applied to the rehabilitation of persons with acquired brain injury, predominantly those with traumatic brain injury and behavioral difficulties. Over time, it has evolved to incorporate aspects of cognitive theory that are seen as fundamental to learning, and there is growing evidence that the approach may be indicated for anyone who cannot independently meet their own social or functional needs (6, 7).

Some of the general principles of neurobehavioral rehabilitation originally described by Wood and Worthington, and which remain relevant (8), include a focus on reducing “social handicap” and improving *social outcomes*, which occurs *post-acutely*, within a *community setting*. It is a *transdisciplinary approach*. The whole multidisciplinary team, which typically comprises neuropsychologists, clinical psychologists, occupational therapists, physiotherapists, and speech and language therapists, jointly focuses on behaviors or outcomes (e.g., becoming able to go out for a meal with family), rather than on improving specific areas of impairment (e.g., reducing social anxiety). The approach recognizes and places centrally the neurological underpinnings of brain injury. For this reason, it is delivered within a *structured environment* which facilitates the consistent application of rehabilitation procedures to promote implicit learning, *consolidation* and *generalization* of habits and routines. The focus on *feedback* and on processes to evaluate outcomes facilitate the development of self-awareness, as well as the adaptation of rehabilitation procedures as the individual progresses. Through interactions with staff during rehabilitation in a variety of settings, patients learn by experience and work toward adjusting to life after brain injury. The key aspects of the approach are informed by neuropsychological principles and psychological formulation. Formulation incorporates how cognition, learning, and emotion together influence the person's adjustment, engagement in rehabilitation, and recovery. It is psychology-led; however, senior members of different clinical professions also contribute to the leadership of services and teams.

Basic behavioral modification principles, for example reinforcement, modeling, feedback, and non-reinforcement, among others, remain key to the rehabilitation programmes across the Trust's rehabilitation centers and hospitals. A unique feature of neurobehavioral programmes is the concept that “every interaction is an opportunity to implement a small building block of the person's rehabilitation”. What that means in practice is that new learning is supported both by members of the transdisciplinary clinical team, who apply these principles in their sessions with patients, and on an everyday, 24/7 principle, by rehabilitation support workers and other floor staff. These floor staff encounter patients many times each day, and apply behavioral strategies, such as, for example, feedback and reinforcement in *every* suitable interaction, which facilitates opportunities for generalization of rehabilitation gains across environments. Furthermore, a novel aspect of the approach, the high frequency possible to achieve through support workers' contacts with patients, further facilitates learning and generalization through repetition. These everyday applications of the approach are guided by the psychology-led transdisciplinary formulations and treatment plans.

Skilling staff to understand and apply these transdisciplinary formulations is a key component of the approach. This is achieved in multiple ways, ranging from formal training on understanding brain injury, to bespoke tutorials introducing specific interventions or clinical guidelines, all developed to support recovery and the achievement of rehabilitation roles. More recently, the Trust has been piloting a new “Rehab Mantras” initiative as a novel way of ensuring the constant embedding of the core principles of the neurobehavioral approach into everyday operation and practice across all our services. This involves identifying and disseminating a “rehab mantra” each month to all staff (e.g. “*Every interaction is rehab*.”), through for example our intranet, email signatures, and screensavers. The engagement and feedback on whether staff are aware of the monthly mantra, and how they have applied it are obtained to monitor learning and skills development. The overarching aim with this initiative is to consolidate an understanding of why rehabilitation is delivered in a certain way, and to ensure consistency in everyday practice. The initiative is also intended to ensure maximum penetration of the model's principles into every aspect of rehabilitation, thus increasing the ‘dose’ of therapeutic interactions by all staff, which is a novel aspect of the NBT approach.

Over time our approach to rehabilitation has evolved. While the term neurobehavioral therapy has been used before—see for example Siegle et al. (9)—here the term means augmenting neurobehavioral brain injury rehabilitation with selected psychotherapy principles and strategies. These are applied both to direct interventions with patients, and to staff training and development, as well as family support. Examples of the latter include Cognitive Behavior Therapy (CBT) and Compassion Focused Therapy (CFT), among others. These provide opportunities to address areas not normally directly covered in “purer” neurobehavioral approaches, including self-awareness and psychological adjustment. Combining targeted psychotherapy principles with a neurobehavioral model to address psychological difficulties pure behavioral approaches fail to achieve, is a further novel aspect of the NBT approach.

This gradual evolution of the original model into the current NBT approach has been driven by various factors, one of which has been type of referrals accepted by different services. An increasing need in the UK to provide more rehabilitation to persons with neurological conditions other than traumatic brain injury, including also the wider clinical population defined as having suffered a stroke (or cerebro-vascular accident; CVA) for example, has had a significant influence on the range of neuropathological conditions seen in the Trust's services. The other factor driving this development, has been the desire to have more complete, integrated multi-disciplinary clinical pathways to meet the needs of patients on the journey from sub-acute, to post-acute, to long-term health and care.

## Methods

### Participants

Data from referrals spanning 2 years, including diagnostic information and routinely collected outcomes, were retrospectively analyzed to assess the effectiveness of the approach.

### Clinical outcome measures

Clinical effectiveness is routinely monitored by periodically analyzing data from global measures of outcome, which provides evidence about the effectiveness of rehabilitation and informs practice development, both clinical and research (10). These routine outcome measures included in this study are:

Supervision Rating Scale [SRS (11)], a 14-point scale that has been developed to assess support needs of those living with a brain injury. Scores can be described into five levels of supervision ranging from 1—independent, to 5—full-time direct supervision.

The BIRT Independent Living Scales—Accommodation and Occupation [BILS, (12)], which comprise two single-item measures. The accommodation scale ranks a person's accommodation placement from one (living independently) to 11 (residing in a secure unit). The occupation scale ranks the level of occupational engagement from one (engaging in competitive academic or work role) to nine (no involvement in regular activities). These scales have been found to correlate well with functional ability and provide a brief method for measuring independent living ability and social participation.

Mayo-Portland Adaptability Inventory—IV [MPAI-4 (13)], is a widely used standardized measure of post-acute outcomes following acquired brain injury. It comprises 29 core items which are grouped into three sub-scales. The Ability Scale focuses on mobility, use of hands, vision, hearing, dizziness, motor speech, verbal and non-verbal communication, attention and concentration, memory, fund of information (including semantic and autobiographical memory; problem-solving and visuo-spatial abilities). The Adjustment scale includes items that measure psychological adjustment following the injury (including for example anxiety, depression, irritability, anger, and self-awareness). The Participation scale comprises items that evaluate social outcomes, including the degree of social contact, initiation, and money management ability. On the MPAI-4 higher scores denote more severe disability.

In addition to these measures, clinicians monitor the progress using a range of methods that are most suited to the person's individual goals, given that goal setting is associated with effectiveness of rehabilitation. Based on a survey of the Trust's psychologists, this usually includes patient feedback (*n* = 9, 81%), observations and behavior monitoring (*n* = 9, 81%), standardized clinical measures (*n* = 7, 64%) and goal achievement scores (*n* = 5, 54%).

### Procedure

This paper describes the overarching Brain Injury Rehabilitation Trust approach, and its clinical implementation, and application to three different ‘streams’ of service provision. These “streams” are illustrated with case vignettes. Quantitative outcomes achieved by patients who completed their programmes over the period between October 2019 and October 2021 were analyzed with R 4.1.2 (14), using descriptive statistics, effect size calculations, and non-parametric tests for ordinal repeated measures and paired samples *t*-tests for standardized scores (i.e., MPAI-4).

## Results

### One approach, three streams

Recent referral data spanning just over 2 years (01 October 2019–31 October 2021) provide more detailed information of the gradual expansion of diagnoses accepted within the Trust's services. These data show that services provide, or have provided (where discharge has taken place), clinical services to persons with diagnoses other than traumatic brain injury, including several types of stroke (15). More specifically, breaking down these data, other neurological diagnoses constituted 20% of the 395 patients admitted during this 2-year period. Some of these diagnoses included Guillain-Barré syndrome, autoimmune conditions, Korsakoff's syndrome, and epilepsy, among others. Table 1 provides summary data of these diagnoses of other neurological conditions.

**Table 1:** Diagnoses for admissions 01 October 2019–31 October 2021.

**Diagnosis**	***N***	**%**
Stroke	182	46%
TBI	136	34%
Other*	25	6%
Hypoxia	21	5%
Infection	15	4%
Toxic injury	14	4%
Neoplasm	2	1%
Total	395	100%

TBI, traumatic brain injury. *Includes Guillain-Barré syndrome, autoimmune conditions, Korsakoff's syndrome, epilepsy, among others.

**Figure 1:**
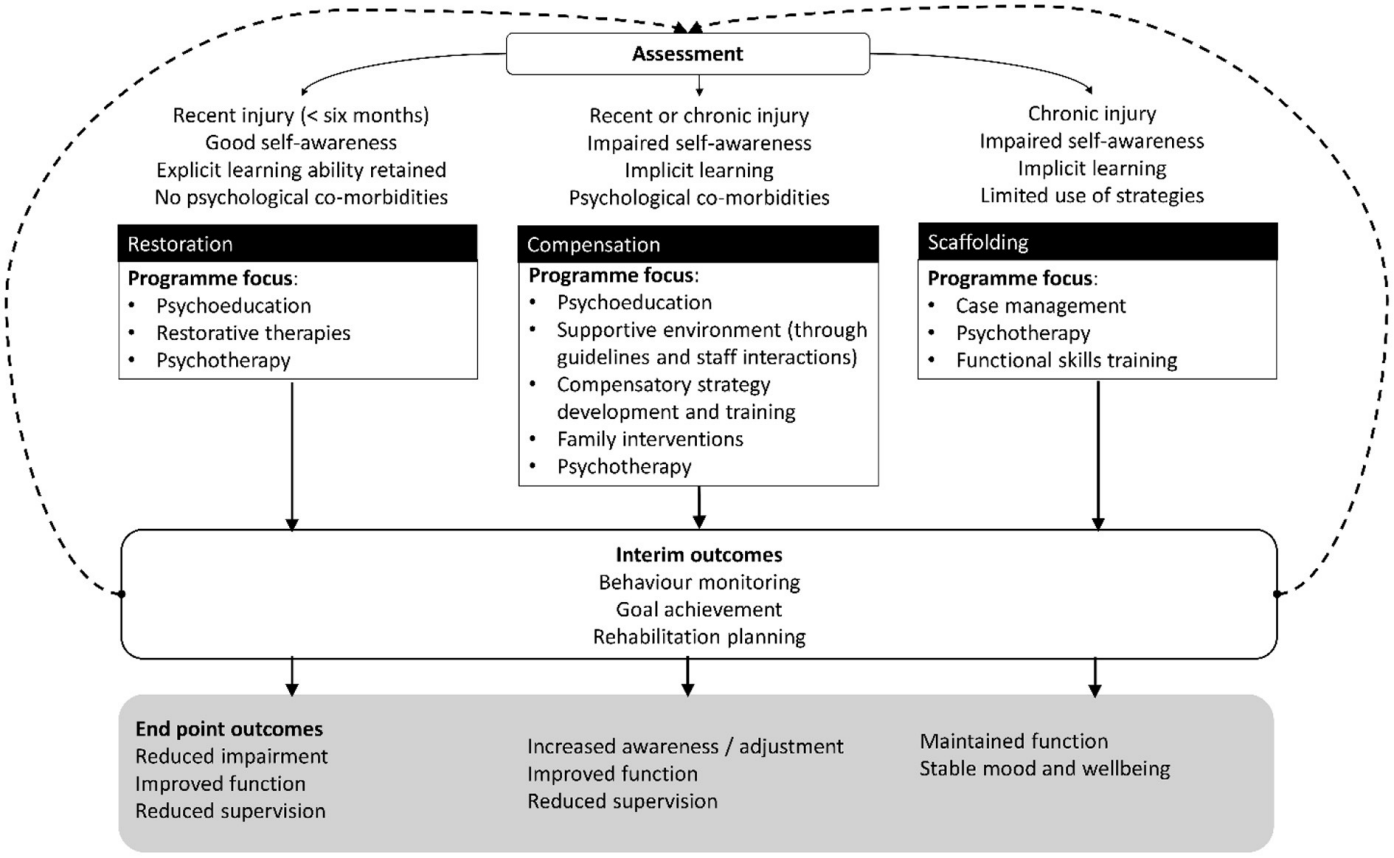
The three clinical streams within the neurobehavioral therapy approach.

Cluster analysis methodology has been used previously to inform and validate the development of three broad streams that match different profiles of clinical need within the NBT approach. This identified specific areas that distinguish between groups, including functioning, neuropathology, comorbidity, and the time since injury or illness onset (16). The findings offered the framework, within the NBT approach, for classifying rehabilitation needs and identifying the best stream for each patient, as shown in Figure 1.

Accordingly, rehabilitation centers or hospitals are now configured and staffed to best meet the different needs of patients who require:

Restoration, which is indicated for people who have significant needs in specific areas, for example self-care, communication, or mobility, because of a recent brain injury, and who are likely to benefit from approaches focused primarily on restoration of function. People who benefit from this stream will usually have good awareness of their injury, and how it has affected them. They also do not show any behaviors of concern (17), which would otherwise prevent them from taking part in rehabilitation (e.g., aggressive behavior, refusal of care and treatment).

Compensation is for people whose needs may present as barriers to taking part in neurorehabilitation, and who are likely to benefit from approaches primarily focused on compensation of function. People in this group may need initial support, prompting, and feedback to become more aware of, and adjust to, the difficulties they face after brain injury. In some cases, they may have both cognitive and emotional difficulties, as well as physical health needs.

Scaffolding and support is for people who are likely to benefit from ongoing clinical input and support to maintain function and prevent relapse or deterioration. Improvements as a result of functional skills training are achievable in this group (18), but this may take longer to come to fruition than the gains seen in those in the restoration or compensation streams.

Data illustrating the three clinical streams are shown in Table 2, summarizing information on diagnoses and other clinical characteristics of patients across these streams for the admissions spanning the aforementioned 2-year period.

**Table 2:** Clinical characteristics across three streams: 01 October 2019–31 October 2021.

**Characteristic**	**Restoration**	**Compensation**	**Scaffolding**
	**(*****N*** **=** **176, 45%)**	**(*****N*** **=** **169, 44%)**	**(*****N*** **=** **43, 11%)**
Distribution of diagnoses	TBI 26%	TBI 43%	TBI 35%
	Stroke 57%	Stroke 40%	Stroke 30%
	Other 17%	Other 17%	Other 35%
Months since injury			
*Me* (*Min*-*Max*)	2 (0–335)	2 (0–273)	4 (0–400)
MPAI-4 (*M, SD*)			
Ability	51 (11)	50 (9)	58 (8)
Adjustment	51 (10)	53 (9)	56 (7)
Participation	56 (12)	55 (11)	64 (9)
Comorbidities			
Schizophrenia	4 (2%)	5 (3%)	0
Drug misuse	10 (6%)	10 (6%)	1 (2%)
Alcohol misuse	23 (13%)	32 (19%)	3 (7%)
Multiple trauma	8 (5%)	7 (4%)	2 (5%)
Other medical conditions	81 (46%)	19 (11%)	4 (9%)

*Stream data were recorded for 388 (98%) of the 395 cases reported on Table 1*.

Three case vignettes containing qualitative information, provide individual examples of these differences.

### Case vignettes

#### Stream 1—Restoration

Mr A was referred for 12 weeks rehabilitation 2 months after suffering a large left cortical ischemic stroke in the middle cerebral artery area. His stay in rehabilitation was later extended by a further 12-weeks due to the COVID-19 pandemic. The first part of his programme focused on comprehensive assessment and management of his cognition and aphasia. The programme then continued with intensive physiotherapy for 45–60 mins a day, 5–6 times a week. This was extended beyond the clinical sessions, through “self-led” exercise sessions where rehabilitation support workers ensured that the exercises were performed correctly within his everyday environment. Mr A also worked on improving problem solving, and motor planning skills with the physiotherapist, occupational therapist and speech and language therapist. In the last few weeks at the unit, the programme focused on stand and walk practice, and on learning exercises to do at home post-discharge. At the end of the programme Mr A was able to walk with a stick and manage his personal care by himself. Mr A's total standardized score on the Mayo-Portland Adaptability Inventory reduced by 12 points to 53, indicating moderate disability at the time of discharge, and clinically robust changes (19). He moved to supported living accommodation as he continued to need help with communicating and carrying out more complex daily tasks, such as cooking and shopping. Despite his cognitive and language difficulties, Mr A was able to participate in intensive rehabilitation from an early stage.

#### Stream 2—Compensation

Ms L was admitted for rehabilitation 15 months after a hypoxic injury resulting from a drug overdose. In Ms L's own words, before the brain injury “*I led quite a chaotic lifestyle*.” She reported struggling with her mood, drinking alcohol, and taking drugs. This eventually led to the overdose that caused her injury. Following discharge from an acute hospital, Ms L needed help with washing, dressing, and eating. She also had memory and vision problems which significantly affected her function and self-confidence. Her rehabilitation programme focused on working with an occupational therapist to relearn how to wash and dress independently and with a psychologist, to learn how to manage her emotions and adjust to her difficulties. Ms L also worked with a speech and language therapist and physiotherapist who developed plans to help her improve communication and achieve her personal fitness goals. She began using a smart speaker to compensate for her memory problems. In preparation for discharge, Ms L spent some time in a transitional rehabilitation flat. Upon discharge, Ms L was able to carry out her personal care independently, including doing her own hair and make up again, something which was important to her, but challenging in the context of her persisting visual impairment. She continued working on her general fitness and health. She reported feeling more confident of herself than when she was first admitted and valuing the things she learned throughout rehabilitation. Ms L went on to live independently in her own home, while continuing to work with community therapists to further her fitness and occupational engagement. She said that while she knew she “*will need support to do a lot of things*” she felt “*able to adapt*.” At this point, Ms L's score on the MPAI-4 had reduced by 14 points to 42, which falls just over the mild limitations range. Ms L's case illustrates how the compensation stream within the NBT approach can be of benefit to those with a complex range of physical, cognitive, and emotional needs. It also illustrates how the use of principles and strategies from psychological therapies can maximize and consolidate the benefits of a “purer” neurobehavioral approach.

#### Stream 3—Scaffolding and support

Mr M was admitted to one of our rehabilitation centers 21 months after sustaining a traumatic brain injury. He had a pre-existing diagnosis of schizophrenia, and after discharge from acute hospital, he had been transferred to a small hospital where he remained for nearly 2 years. The aim of the post-acute neurobehavioral therapy programme was to enable him to move into a more independent living arrangement. The initial focus of the programme was on sleep management. Mr M was sleeping as little as 2 h per night when he arrived at the service. This involved monitoring fluid intake, helping him change his caffeinated drink intake habits, and developing an engagement programme of activities that would discourage daytime sleeping and re-calibrate bedtimes to allow an 8-h sleeping window. Mr M also attended mindfulness and relaxation groups with other patients and engaged in cognitive training to help with his problem solving and speech and language therapy for his communication difficulties. He had regular gym sessions with physiotherapists. After several weeks, Mr M was able to sleep around 7 h each night. He then started working with an occupational therapist to improve his ability to independently prepare basic meals, which was implemented using errorless learning. The training sessions were filmed so that the occupational therapist could monitor how much support was given and the type of assistance that was needed. At the end of the training, the number of errors per session had reduced from 23 to three, and the time required for meal preparation from 90 to 20 mins. Mr L was discharged into supported living accommodation for people with brain injuries, albeit with significantly reduced care needs. Living in a supported living environment is expected to enable Mr L to maintain the gains he made during inpatient rehabilitation, while preventing potential relapse of his pre-existing condition, which he could not manage independently. Change scores on the MPAI-4 were not available, however, Table 3 shows results for a sample of 28 people served within the same stream.

**Table 3:** Outcomes upon discharge from rehabilitation by clinical stream.

	**Restoration**		**Compensation**		**Scaffolding**	
	***N*** **= 118**		***N*** **= 101**		***N*** **= 28**	
Weeks in service*	12 (13)		14 (14)		35 (25)	
	A	D	A	D	A	D
Supervision rating scale*	8 (5)	4 (6)	11 (3)	4 (7)	10 (3)	8 (1)
BILS accommodation*	9 (0)	5 (4)	9 (0)	5 (5)	9 (0)	8 (3)
BILS occupation*	9 (1)	8 (2)	9 (0)	8 (2)	9 (5)	8 (1)
MPAI-4 ability^‡^	52 (10)	45 (12)	50 (10)	44 (10)	56 (5)	54 (7)
MPI-4 adjustment^‡^	51 (8)	45 (12)	54 (8)	49 (11)	55 (6)	54 (11)
MPAI-4 participation^‡^	56 (11)	48 (12)	55 (11)	50 (11)	65 (8)	57 (11)
*N (%)* patients showing change over the MPAI-4 MCI threshold (13)	76 (64%)		55 (54%)		12 (43%)	

A, admission; D, discharge; *Non-parametric variable, results denote Me (IQR); ^‡^Parametric variable, results denote M (SD).

In practice there may be overlap, or indeed patients moving between streams, either due to recovery, or to new symptoms, but in theory the three streams map onto broader clinical pathways in brain injury rehabilitation, whilst simultaneously also capturing other diagnostic groups (e.g., neoplasms, anoxia, and brain infections).

#### Effectiveness of the approach

Table 3 shows the length of stay in service, scores on each measure on admission and discharge, as well as the proportion of people showing improvements above the clinically significant threshold on the MPAI-4 (19).

The median scores on the SRS showed a statistically significant reduction of supervision needs (*W* = 44,991, *p* < 0.001), which was large in the Restoration and Compensation streams, and medium within the Scaffolding stream. Comparable changes were found on the BILS Accommodation (*W* = 49,356, *p* < 0.001), indicating discharges into less structured community-based settings (e.g., from nursing home or hospital on admission to living in own home with or without support). Changes in occupation were more modest, but meaningful and significant (*W* = 49,356, *p* < 0.001), with 28% of the people discharged into an independent living setting engaged in an occupational role. The MPAI-4 scores revealed an overall significant reduction of disability in all domains of the MPAI-4 [Abilities: *t*_(485)_ = 6.58, *p* < 0.01; Adjustment *t*_(458)_ = 5.39, *p* < 0.01; Participation *t*_(489)_ = 5.45, *p* < 0.01], but these positive changes were generally limited to improvements in social participation for those in the Scaffolding and support stream.

## Discussion

This paper described the neurorehabilitation approach followed within a UK charitable rehabilitation provider, the evolution of the approach, and outcome data to support the effectiveness of the evolved approach. The data reported here showed that more patients in early rehabilitation achieved meaningful outcomes. Early, more intense neurorehabilitation has been found to be effective by other authors too, for example Königs et al. (20). The approach described in this paper, provides rehabilitation across the long-term clinical pathway, in an attempt to address unmet need. Patients with brain injury have limited access to rehabilitation, and in particular as regards psychology (21). Psychological input is central to the NBT approach to rehabilitation.

Determining the effectiveness of neurorehabilitation across the whole, long-term clinical pathway is very complex, and not every question in this regard can be answered by, for example, prospective randomized control trials [RCTs (22)]. Future research could make use of retrospective analyses of ‘big data’, for example, as collected by census. Future studies may also use prospective designs to follow the same patients longitudinally across the long-term rehabilitation journey in services which provide such a pathway and compare to patients who only receive one component of rehabilitation, adding to our understanding of this factor’s impact on outcomes (23).

### Limitations

There are some limitations inherent to the data reported. The first is that these data are based on clinicians' judgement, and do not always directly reflect the patients' or family views of outcomes. To improve this, a global impression of change measure has been included in the outcome measures system, but insufficient data were available for analysis at the time of writing (3%). Furthermore, individuals who were discharged earlier than planned (21% of the total discharged), were excluded from the analyses, as they would not have completed their rehabilitation programme. However, the data did include results for individuals who were discharged later than planned, which has been found to influence outcomes (24). Delays in discharges is another factor recently added to routine data capture, with the aim of further investigating the effects this may have on outcomes. Finally, while the clinical streams go some way to matching the rehabilitation programme to key patient characteristics, the more detailed contents of each individual programme will vary. This means that sensitivity to change may be reduced when using overall scores on global measures like the MPAI-4, as people will engage in interventions to target some areas but not others. At present, it is difficult to account for this individual variation in the analyses of outcomes within each stream.

### Strengths

One of the main strengths of this paper is that it describes in detail a modified neurobehavioral approach, which may offer opportunities to develop rehabilitation programmes that lend themselves to addressing a wider (than behavior) range of difficulties patients may present. The outcome data reported here suggest that it is possible to achieve meaningful change with such modified programmes. The data provide preliminary evidence that treatments in the three streams of rehabilitation described can potentially benefit a wider group of patients with neurological injury or illness, over the longer-term journey of a more complete clinical pathway.

## Data Availability Statement

The data analyzed in this study is subject to the following licenses/restrictions: access to data analyses can be requested from SR. Requests to access these datasets should be directed to sara.dasilvaramos@thedtgroup.org.

## Ethics Statement

Ethical review and approval was not required for the study on human participants in accordance with the local legislation and institutional requirements. Written informed consent for participation was not required for this study in accordance with the national legislation and the institutional requirements. Written informed consent was obtained from the individual(s) for the publication of any potentially identifiable images or data included in this article.

## Author contributions

RC defined the topic, aims, scope of the paper, and wrote the first draft. SR conducted the statistical analysis, data presentation, sourced, and wrote up the case vignettes. Both authors contributed to the writing and development of subsequent drafts and approved the final version of the paper.

## Funding

The authors are grateful for the financial support received from their employer, the Disabilities Trust, for open access publishing of this paper.

## Conflict of interest

The authors declare that the research was conducted in the absence of any commercial or financial relationships that could be construed as a potential conflict of interest.

## Publisher's note

All claims expressed in this article are solely those of the authors and do not necessarily represent those of their affiliated organizations, or those of the publisher, the editors and the reviewers. Any product that may be evaluated in this article, or claim that may be made by its manufacturer, is not guaranteed or endorsed by the publisher.

## References

[1] Moore W. The Edwin smith papyrus. BMJ. (2011) 342:d1598. 10.1136/bmj.d1598

[2] Wilson BA Winegardner J van Heugten CM Ownsworth T. Neuropsychological Rehabilitation: The International Handbook. Milton Park: Routledge (2017). 10.4324/9781315629537

[3] Hebb D. The Organization of Behavior. New York: Wiley (1949).

[4] Gresham GE Stason WB Duncan PW. Post-Stroke Rehabilitation. Rockville, Maryland: US Department of Health and Human Services, Agency for Healthcare Policy and Research (2004).

[5] Wilson BA. Cognitive rehabilitation: how it is and how it might be. J Int Neuropsychol Soc. (1997) 3:487–96. 9322409

[6] Wood RL Worthington AD. Neurobehavioural rehabilitation: a conceptual paradigm. In: Neurobehavioural Disability and Social Handicap Following Traumatic Brain Injury, eds R. L. Wood and T. M. McMillan (Hove: Psychology Press) (2001).

[7] Cattelani R Zettin M Zoccolotti P. Rehabilitation treatments for adults with behavioral and psychosocial disorders following acquired brain injury: a systematic review. Neuropsychol Rev. (2010) 20:52–85. 10.1007/s11065-009-9125-y 20143264

[8] Worthington A Alderman N. Neurobehavioural rehabilitation: a developing paradigm. In: McMillan TM, Wood RLl, editors. Leiden: Psychology Press (2017). 10.4324/9781315684710-2

[9] Siegle GJ Ghinassi F Thase ME. Neurobehavioral therapies in the 21st century: summary of an emerging field and an extended example of cognitive control training for depression. Cognit Ther Res. (2007) 31:235–62. 10.1007/s10608-006-9118-6

[10] Freeman JA Hobart JC Playford ED Undy B Thompson AJ. Evaluating neurorehabilitation: lessons from routine data collection. J Neurol Neurosurg Psychiatry. (2005) 76:723–8. 10.1136/jnnp.2004.035956 15834035 PMC1739616

[11] Boake C. Supervision rating scale: a measure of functional outcome from brain injury. Arch Phys Med Rehabil. (1996) 77:765–72. 8702369 10.1016/s0003-9993(96)90254-3

[12] Ramos SDS Oddy M Haye L Goodson A. Preliminary investigation of the reliability and validity of the BIRT independent living scale. Disabil Rehabil. (2017) 40:2817–23. 10.1080/09638288.2017.1362594 28805087

[13] Malec J Lezak M Malec J Lezak M. MPAI-4 manual. (2003) 2003:1–84. 10.1016/j.apmr.2003.08.004

[14] R Core Team. R: A Language Environment for Statistical Computing. Vienna: R Foundation for Statistical Computing (2021). Available online at: https://www.R-project.org/ (accessed July 11, 2022).

[15] Bamford J Sandercock P Dennis M Warlow C Burn J. Classification and natural history of clinically identifiable subtypes of cerebral infarction. Lancet. (1991) 337:1521–6. 1675378 10.1016/0140-6736(91)93206-o

[16] Ramos SDS Copstick S. Can cluster analysis help us better plan, communicate, and deliver brain injury rehabilitation? In: Proceedings of the 13th World Congress on Brain Injury Toronto (2019).

[17] Banks R Bush A Baker P Bradshaw J Carpenter P Deb S. Challenging Behaviour: a Unified Approach. Royal College of Psychiatrists, British Psychological Society and Royal College of Speech and Language Therapists. London: Royal College of Psychiatrists (2007). p. 1–79.

[18] Parish L Oddy M. Efficacy of rehabilitation for functional skills more than 10 years after extremely severe brain injury. Neuropsychol Rehabil. (2007) 17:1–13. 10.1080/09602010600750675 17454695

[19] Malec JF Kean J Monahan PO. The minimal clinically important difference for the mayo-Portland adaptability inventory. J Head Trauma Rehabil. (2017) 32:E47–54. 10.1097/HTR.0000000000000268 28489702 PMC5432408

[20] Königs M Beurskens EA Snoep L Scherder EJ Oosterlaan J. Effects of timing and intensity of neurorehabilitation on functional outcome after traumatic brain injury: a systematic review and meta-analysis. Arch Phys Med Rehabil. (2018) 99:1149–59.e1. 10.1016/j.apmr.2018.01.013 29428344

[21] Andelic N Røe C Tenovuo O Azouvi P Dawes H Majdan M. Unmet rehabilitation needs after traumatic brain injury across Europe: results from the CENTER-TBI study. J Clin Med. (2021) 10:1035. 10.3390/jcm10051035 33802336 PMC7959119

[22] Turner-Stokes L Nair A Sedki I Disler PB Wade DT. Multi-disciplinary rehabilitation for acquired brain injury in adults of working age. Cochrane Database Syst Rev. (2005) 1:CD004170. 10.1002/14651858.CD004170.pub2 16034923

[23] Andelic N Ye J Tornas S Roe C Lu J Bautz-Holter E. Cost-effectiveness analysis of an early-initiated, continuous chain of rehabilitation after severe traumatic brain injury. J Neurotrauma. (2014) 31:1313–20. 10.1089/neu.2013.3292 24720788

[24] Worthington AD Oldham JB. Delayed discharge from rehabilitation after brain injury. Clin Rehabil. (2006) 20:79–82. 10.1191/0269215506cr881oa 16502753

